# Human papillomavirus-mediated cervical cancer: epigenetic interplay and clinical implications

**DOI:** 10.3389/fmicb.2025.1633283

**Published:** 2025-10-02

**Authors:** Sandeep Sisodiya, Payal Singh, Tannu Joshi, Mehreen Aftab, Nasera Firdausi, Asiya Khan, Neetu Mishra, Nida Jamil Khan, Pranay Tanwar, Vivek Gupta, Showket Hussain

**Affiliations:** ^1^Cellular and Molecular Diagnostics (Molecular Biology Group), ICMR-National Institute of Cancer Prevention and Research, Noida, India; ^2^Symbiosis School of Biological Sciences, Symbiosis International (Deemed University) (SIU), Pune, India; ^3^Department of Bioscience, Jamia Millia Islamia, New Delhi, India; ^4^Laboratory Oncology Unit, Dr. BRA-IRCH, All India Institute of Medical Sciences, New Delhi, India; ^5^Department of Pathology, Government Institute of Medical Sciences, Greater Noida, India

**Keywords:** cervical cancer, human papillomavirus, epigenetics, screening, biomarker

## Abstract

Cervical cancer is a one of the leading causes of mortality in women, and WHO’s initiative to eliminate cervical cancer by 2030 needs to explore several emerging research areas for its elimination such as epigenetics which could play a crucial important role in the cervical cancer pathogenesis driven by persistent high-risk-human papillomavirus infection. Understanding the molecular and epigenetic mechanisms underlying HPV infection and its progression to cancer is critical for advancing prevention, diagnosis, and treatment strategies, which may play a crucial role in eliminating cervical cancer. Persistent infection of human Papillomavirus is intricately linked to the initiation and progression of cervical cancer with different molecular mechanisms, pathways, viral genes, and proteins. HPV-mediated alterations in the host epigenome play a pivotal role in driving oncogenic transformation by modulating gene expression, chromatin dynamics, and DNA methylation patterns, ultimately disrupting normal cellular functions. The relationship between HPV-induced epigenetic changes and cancer progression underscores the virus’s ability to bypass conventional gene-silencing mechanisms. By altering critical regulatory pathways, HPV not only fosters cancerous growth but also influences patient responses to existing treatments, posing challenges to effective disease management. In this current review, we have discussed the role of epigenetic disruptions caused by HPV, which provided a unique opportunity to identify novel therapeutic targets and biomarkers. Epigenetic factors, being reversible and independent of direct genetic manipulation, offer promising avenues for innovative drug delivery strategies. Such approaches could enhance disease management by advancing therapeutic strategies and diagnostics for improving patient outcomes.

## 1 Introduction

Cervical cancer (CC) remains one of the leading causes of cancer-related deaths, especially in low-and middle-income countries (LMICs), and also 4th most common cancer in women globally. It is well-established that cervical cancer occurs due to persistent infection of high-risk human papillomavirus (HR-HPV); among all HR-HPV, HPV 16 and 18 are responsible for most of the cervical cancers (Khan et al., 2022). Although HPV infection usually goes away on its own, in some cases, it stays in the body and can lead to cancer due to other cellular, molecular mechanisms, and genetic and epigenetic alterations, which are crucial contributors to cervical carcinogenesis (Neyaz et al., 2008).

**Figure 1:**
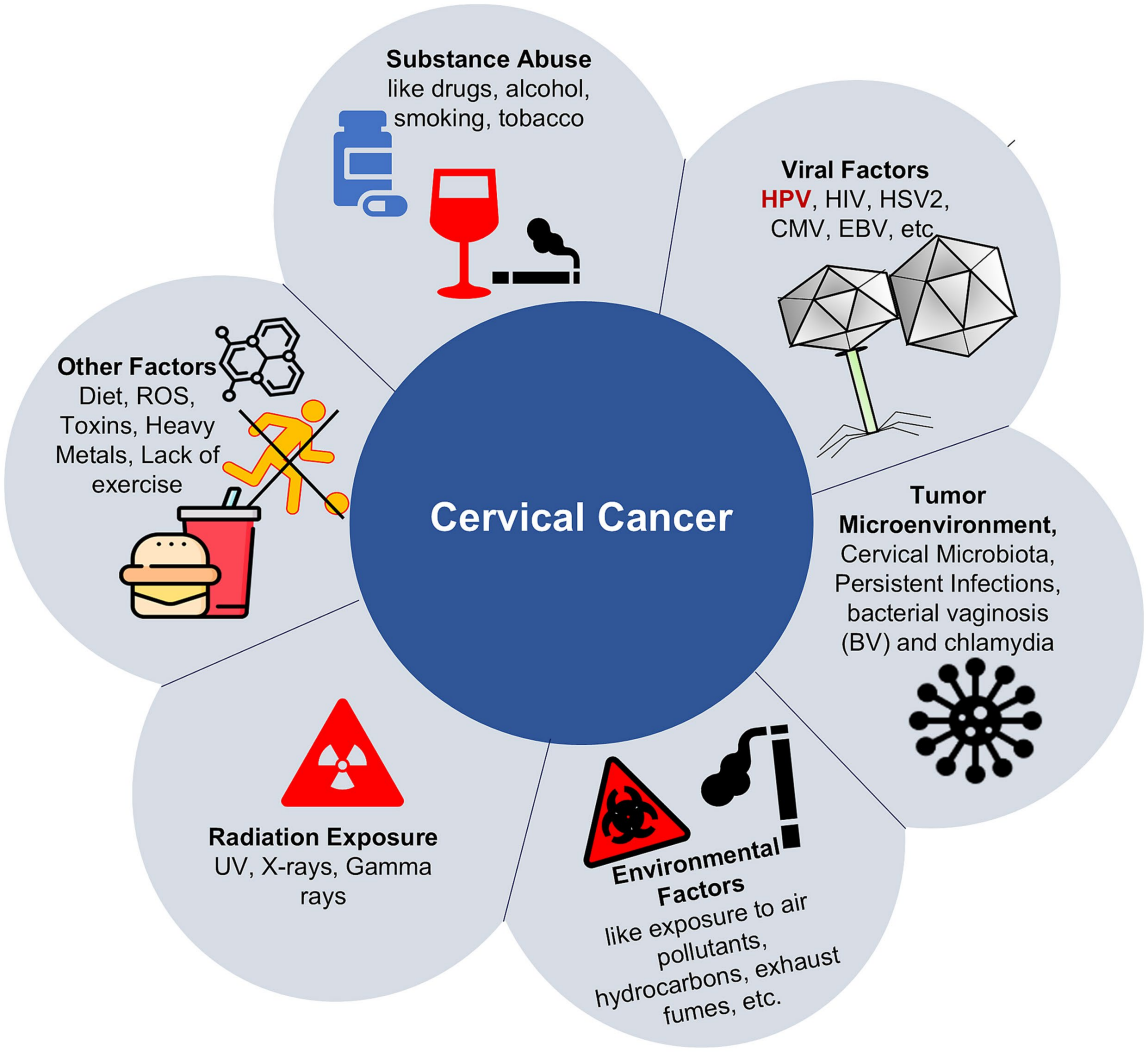
Representing causes of epigenetic alterations, leading to cervical cancer.

Epigenetics involves the regulation of gene expression through heritable modifications that do not alter the underlying DNA sequence (Hamilton, 2011; Gibney and Nolan, 2010; Dasari et al., 2015; Hanahan, 2022). There are several factors that influence epigenetic changes, such as diet, exposure to toxins, psychological stress, and smoking (Figure 1). This involves several epigenetic mechanisms, such as DNA methylation, histone modifications, chromatin remodeling, and regulation by non-coding RNAs, all of which are essential for controlling gene expression and preserving genomic stability. When these processes become dysregulated, these alterations can result in abnormal gene expression, contributing significantly to the initiation and progression of cancer (Hussain et al., 2022; Sobti et al., 2011).

Epigenetic alterations, including global DNA hypomethylation, tumor suppressor gene hypermethylation, and histone modifications, occur throughout cervical carcinogenesis in both HPV and host genomes. Establishment of HPV shows that it exploits the host’s epigenetic machinery to promote viral persistence, evade immune surveillance, and drive oncogenic transformation. The viral oncoproteins E6 and E7 are central players in this process, as they not only disrupt tumor suppressor pathways, notably p53 and Rb, but also modulate the host epigenetic landscape to favor malignancy (Da Silva et al., 2021).

Aberrant DNA methylation is among the earliest and most extensively investigated epigenetic modifications in HPV-associated cervical cancer. This modification involves attaching a methyl group to the 5-carbon position of cytosine bases located within CpG dinucleotide sequences, particularly in promoter regions, often resulting in transcriptional repression of the associated genes. In cervical cancer, hypermethylation of tumor suppressor gene promoters such as CDKN2A, DAPK1, RASSF1A, CADM1, and MAL has been consistently observed. These alterations result in the silencing of critical genes involved in apoptosis, cell cycle regulation, and cell adhesion, thereby promoting carcinogenesis (Zhu et al., 2020). Simultaneously, global hypomethylation, especially in repetitive elements and oncogenes, contributes to genomic instability and aberrant activation of proto-oncogenes. The E7 oncoprotein has been shown to upregulate DNA methyltransferases (DNMTs), particularly DNMT1 and DNMT3b, which mediate these methylation changes. E6, through degradation of p53, also indirectly influences DNA methylation and histone modification pathways, underscoring the cooperative roles of these viral proteins in epigenetic reprogramming (Sen et al., 2018).

**Figure 2:**
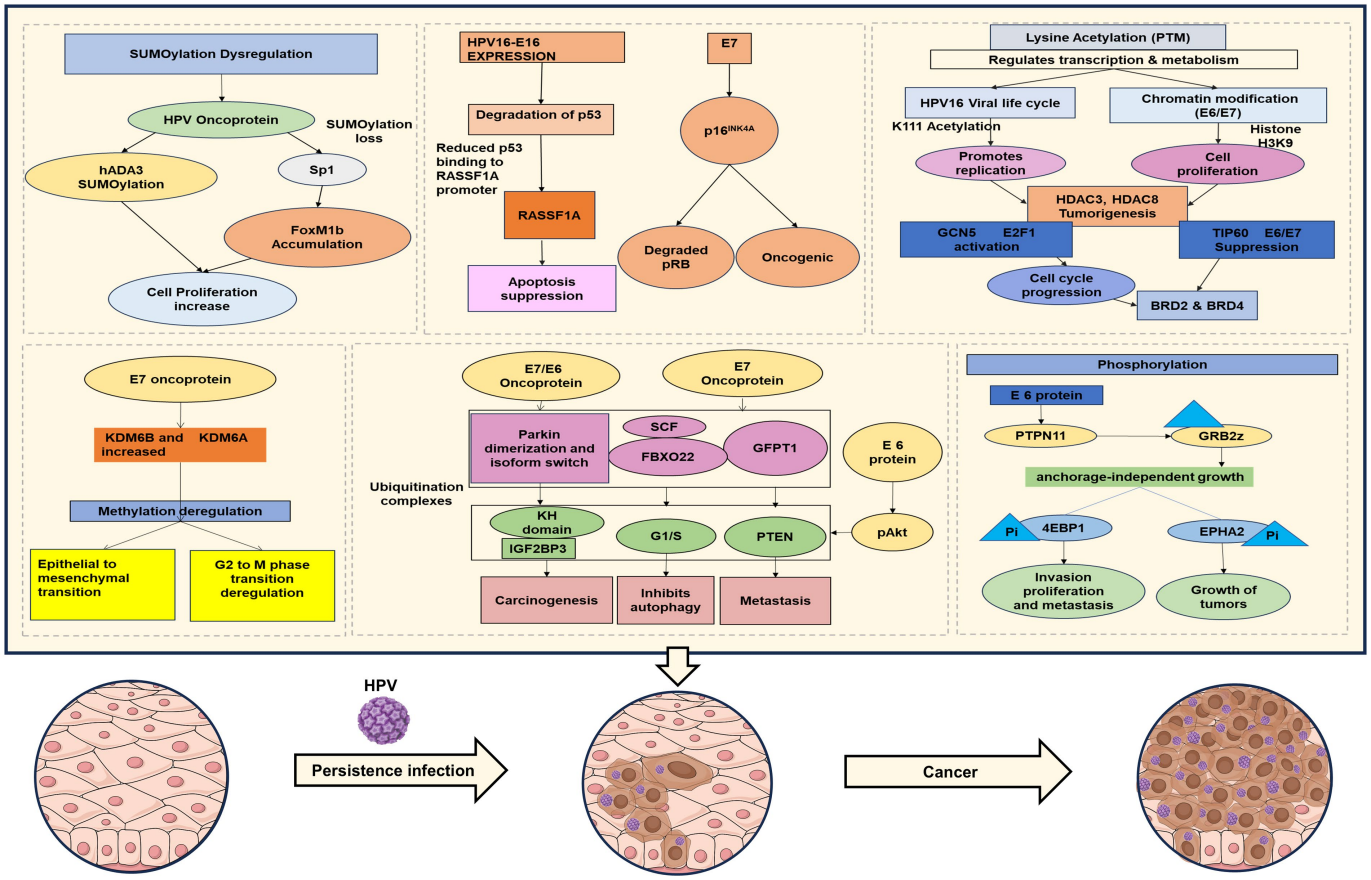
Overview of epigenetic changes/factors contributing to HPV-associated cervical cancer pathogenesis.

An important epigenetic mechanism centers on histone proteins, which forms the structural framework around which the DNA is wrapped. These histones are subject to a range of post-translational modifications, such as acetylation, methylation, phosphorylation, and ubiquitination, that collectively regulate chromatin architecture and gene expression (Figure 2). These changes can either stimulate or suppress gene transcription depending on the nature and position of the modifications. In cervical cancer, aberrant histone modification patterns are frequently observed and are often mediated by the deregulation of histone-modifying enzymes (Zhao and Shilatifard, 2019). HPV oncoproteins have been found to interact with histone acetyltransferases (HATs) and histone deacetylases (HDACs), leading to an imbalance in acetylation patterns that influence gene expression. For example, E7 has been shown to inhibit HDAC activity, thereby promoting the expression of cell cycle-related genes. Additionally, the HPV E6 oncoprotein has been shown to interact with the histone methyltransferase EZH2, leading to increased trimethylation of histone H3 at lysine 27 (H3K27me3), a repressive epigenetic mark linked to gene silencing. This mechanism contributes to the epigenetic inactivation of crucial tumor suppressor genes and regulatory pathways, thereby promoting the progression of cervical carcinogenesis (Lourenco de Freitas et al., 2020).

Non-coding RNAs (ncRNAs), particularly microRNAs (miRNAs) and long non-coding RNAs (lncRNAs), are increasingly recognized as crucial epigenetic regulators in the development and progression of cervical cancer. These RNAs do not encode proteins but regulate gene expression post-transcriptionally and via chromatin remodeling complexes. HPV infection alters the expression of several ncRNAs, which in turn modulate pathways critical to cell proliferation, apoptosis, and immune response (Hussain et al., 2022; Sharma and Munger, 2020). MiRNAs such as miR-34a, miR-203, and miR-375 are often downregulated in HPV-positive cervical cancers, either through direct targeting by HPV oncoproteins or through promoter hypermethylation. Conversely, oncogenic miRNAs like miR-21 are upregulated and contribute to tumor progression LncRNAs, such as HOTAIR, MALAT1, and PVT1, are also dysregulated in HPV-associated cervical cancer and function by recruiting epigenetic modifiers to specific genomic loci, thereby altering chromatin states and gene expression (Gomez-Gomez et al., 2013; Jiang et al., 2014; Zhou et al., 2021; Cho et al., 2018).

The interplay between HPV infection and host epigenetic machinery is central to the development and progression of cervical cancer. HPV-induced epigenetic reprogramming drives the silencing of tumor suppressor genes, activation of oncogenes, and disruption of normal cellular pathways, culminating in malignant transformation, and understanding these changes is crucial for future cervical cancer management. The aim of this current review is to discuss the role of epigenetics and its clinical implications in HPV-mediated cervical cancer, focusing on molecular epigenetic modifications, diagnostics/screening, and therapeutic strategies in cervical cancer and its future prospects.

## 2 Epigenetic mechanism and an overview of HPV mediated cervical cancer

Over the past few decades, extensive research into the natural history of cervical cancer has established that persistent infection with certain high-risk human papillomavirus (HPV) types is the primary etiological factor driving the disease’s development (Chan et al., 2019). Since the early 1980s, a strong and well-documented association has been established between human papillomavirus (HPV) infection and cervical squamous cell carcinoma. Notably, the strength of this association surpasses that observed between tobacco use and lung cancer (Chan et al., 2019). Around 30 sexually transmitted HPV types have been identified that can primarily infect the cervix, vagina, vulva, penis, and anus. Notably, one or more of these types are found in 99.7% of cervical squamous cell carcinoma cases, highlighting their crucial role in the pathogenesis of the disease (Chan et al., 2019; Walboomers et al., 1999).

High-risk human papillomavirus (HPV) types, especially HPV16 and HPV18, play a pivotal role in the development of cervical cancer, primarily through the oncogenic functions of their E6 and E7 proteins. HPV comprises a group of genetically related viruses, each classified based on nucleotide sequence variations and assigned a type number corresponding to the order of identification. More than 200 HPV types are known to exist (de Villiers et al., 2004) and Among all, approximately 15 are strongly linked to the development of cervical cancer. Genital HPV types are broadly categorized into high-risk (oncogenic) and low-risk (non-oncogenic) groups based on their potential to cause cervical malignancies and related precursor lesions. Low-risk types, such as HPV 6, 11, 42, 43, and 44, are typically associated with benign conditions like genital warts, whereas high-risk types—including HPV 16, 18, 31, 33, 35, 39, 45, 51, 52, 56, 58, 59, 68, 73, and 82—are implicated in cervical cancer and its premalignant stages (Walboomers et al., 1999; Unger and Barr, 2004). Although low-risk HPV subtypes are primarily associated with benign lesions, they have occasionally been detected in cervical carcinoma cases. HPV typically targets the mucocutaneous epithelium, where it replicates in differentiated epithelial cells. This infection can interfere with normal cell-cycle regulation, promoting unrestrained cell proliferation and contributing to the accumulation of genetic alterations (Unger and Barr, 2004; Okunade, 2020). HPV infection has been implicated in nearly 90% of cervical adenocarcinomas occurring in women under the age of 40, whereas its presence drops significantly to approximately 43% in adenocarcinoma cases among women aged 60 and above (Burd, 2003).

Nonetheless, several additional factors can influence an individual’s capacity to eliminate HPV infection. These include genetic susceptibility, such as polymorphisms in major histocompatibility complex (MHC) genes and specific variants in the p53 gene implicated in HPV persistence and clearance, as well as genetic diversity among HPV types, co-infection with multiple HPV strains, reinfection frequency, hormonal influences, and the host immune response. Thus, while the presence of high-risk HPV is a critical prerequisite, it alone may not be sufficient for cervical cancer development. The progression to malignancy depends on the interplay between high-risk HPV types and various cofactors that modulate viral oncogenic activity. These modifiers include: Suppressed primary immune response, long-term use of oral contraceptives, cigarette smoking, and increasing parity (Okunade, 2020). Apart from this, persistent infection with high-risk HPV types plays a crucial role in cervical carcinogenesis by triggering epigenetic modifications in both the viral and host genomes. These alterations, including changes in DNA methylation patterns, histone modifications, and dysregulation of non-coding RNA (ncRNA) expression, result in the activation of oncogenes or the silencing of tumor suppressor genes, thereby facilitating the progression toward cervical cancer (Figueroa-Angulo et al., 2025).

### 2.1 Methylation

DNA methylation is a widely researched epigenetic modification in mammals. In healthy cells, it plays a key role in regulating gene expression and maintaining stable gene silencing. It works in conjunction with histone modifications, and their interaction is vital for genome regulation by altering chromatin structure (Kulis and Esteller, 2010). Methylation involves the enzymatic addition of a methyl group to the 5′ positions of cytosine, using S-adenosyl-methionine as the methyl donor, a process catalyzed by DNA methyltransferases DNMT1, DNMT3a, and DNMT3b (Kulis and Esteller, 2010; Tsai and Baylin, 2011). In the mammalian genome, methylation predominantly occurs at cytosines within CpG dinucleotides, with a small percentage of these dinucleotides clustered in regions known as CpG islands. These islands are often associated with gene promoters, transcription start sites, and/or first exons. In cancer, two major altered methylation patterns are observed: global DNA hypomethylation and promoter DNA hypermethylation (Tsai and Baylin, 2011). Hypermethylation, observed across nearly all tumor types, is often confined to CpG islands in gene regions, whereas hypomethylation frequently affects repeated DNA sequences, retrotransposons, endogenous retroviral elements, and unique transcription control sequences, suggesting its independent role in cancer development and progression (Esteller, 2002; Ehrlich, 2002). Epigenetic silencing of genes impacts key pathways involved in cancer initiation, progression, invasion, and metastasis, including cervical cancer. One of the pioneering works done by F Rösl, A. Arab, et al., suggested that DNA methylation is a stringent regulatory pathway for modulation of HPV gene expression (Rosl et al., 1993).

### 2.2 Histone modification and chromatin remodeling

Within cells, DNA is organized into chromatin, a highly dynamic structure in which the nucleosome serves as the fundamental unit of packaging. Histones constitute the core of the nucleosome, which is composed of an octamer containing the four core histone proteins—H3, H4, H2A, and H2B—around which approximately 147 base pairs of DNA are tightly wrapped. Each of these core histones possesses an N-terminal tail that is abundant in positively charged lysine and arginine residues. These tails are subject to various covalent post-translational modifications (PTMs), which play a coordinated role in regulating chromatin structure and function. Some modifications can influence the electrostatic interactions between histones and DNA, thereby affecting chromatin compaction and gene transcription. Additionally, certain PTMs act as docking sites for chromatin-binding proteins, which may further mediate changes in chromatin dynamics and gene regulation. Dysregulation of these chromatin-associated factors has been implicated in multiple cancers, including cervical cancer, where aberrant histone modifications and the malfunction of components like the SWI/SNF complex subunits BAF47 and BAF250A have been associated with tumorigenesis (Tsai and Baylin, 2011).

Histone modifications influence chromatin compaction, nucleosome dynamics, and transcription by altering DNA accessibility and facilitating the recruitment of DNA-binding proteins, thereby regulating gene expression. Post-translational modifications (PTMs) of histones serve as a diverse and flexible set of epigenetic marks, playing crucial roles not only in dynamic cellular processes like transcription regulation and DNA repair, but also in the long-term maintenance of repressive chromatin states (Audia and Campbell, 2016). This intricate regulatory system is coordinated by three main groups of proteins: “writers,” which add histone modifications; “readers,” which recognize and interpret these marks; and “erasers,” which remove them. The reversibility of these modifications contributes to the plasticity of the genome, enabling dynamic control of gene expression (Gomez-Gomez et al., 2013). Histone deacetylase inhibitors (HDACis) function by inhibiting the removal of acetyl groups from histones, thereby enhancing histone acetylation levels. This loosens the interaction between DNA and histones, facilitating a more relaxed chromatin structure, and as a result, contributes to the regulation of gene expression (de Villiers et al., 2004).

There are several types of modifications that occur, such as SUMOylation, a type of post-translational modification categorized by the covalent bonds of Small Ubiquitin-like Modifier (SUMO) proteins to specific target proteins, which has gained recognition as a key regulatory mechanism in cancer. It influences the activity of cell cycle regulators, oncogenes, and tumor suppressor genes, thereby impacting tumor development and progression. The dynamic changes in SUMOylation throughout the cell cycle are maintained by precise spatial and temporal control of the SUMO machinery. Moreover, SUMOylation works in concert with other post-translational modifications to support proper cell cycle advancement. Disruption of SUMOylation or deSUMOylation enzymes can lead to significant impairments in cell proliferation, genome stability (Eifler and Vertegaal, 2015) DNA damage response (Jackson and Durocher, 2013) and protein trafficking (Melchior et al., 2003).

Acetylation is a post-translational modification and is reversible in nature. It targets lysine residues across a broad range of proteins, which play a crucial role in modulating cellular plasticity. While approximately 80% of human proteins can undergo N-terminal acetylation, its most profound effects are seen in histone modifications, where it directly impacts gene regulation (Lopez-Banuelos and Vega, 2022). While acetylation was initially identified as a major post-translational modification (PTM) in histones, more recent research has shown that acetylation of non-histone proteins also plays a critical role in regulating protein–protein and protein-DNA interactions, influencing protein localization, stability, and functional activation (Narita et al., 2019). The acetylation of non-histone proteins plays a vital role in various cellular functions, including gene expression, metabolism, and the processes of DNA replication and repair. It also governs cell cycle progression by influencing nutrient uptake, initiating signaling pathways, activating transcription factors, and regulating checkpoints that determine whether the cell cycle continues or is arrested (Lopez-Banuelos and Vega, 2022; Narita et al., 2019).

Functionally, acetylation of lysine residues on histone tails reduces chromatin compaction by neutralizing the positive charge of lysine. This weakens the electrostatic attraction between the negatively charged DNA and histones, resulting in a more open chromatin structure. Histone acetylation is closely linked to transcriptional activation, particularly at enhancers, promoters, and within gene bodies, where it facilitates access to transcriptional machinery (Di Cerbo and Schneider, 2013). Altered histone acetylation levels, particularly at lysine 16 on histone H4, are linked to cancer phenotypes. Hyperacetylation of proto-oncogenes may lead to their activation or altered regulation of gene expression, whereas hypoacetylation of tumor suppressor genes, particularly at promoter regions, is often associated with DNA methylation, contributing to the transcriptional repression process. Enzymes known as lysine acetyltransferases (KATs), also referred to as histone acetyltransferases (HATs), catalyze the addition of acetyl groups to both histone and non-histone proteins such as p53, Rb, and MYC. In contrast, histone deacetylases (HDACs) remove these acetyl groups, thereby influencing gene expression and chromatin structure. Both HAT and HDAC activities are crucial for proper gene expression regulation, and any changes in their expression or function can impact chromatin regulation.

Apart from this, phosphorylation and ubiquitination are also a part of these histone/chromatin modifications. Phosphorylation is one of the key post-translational modifications in which phosphate groups are added to proteins by specific kinases, playing an essential role in controlling various cellular processes such as cell cycle progression, apoptosis, and signal transduction. The ubiquitination process involves the attachment of ubiquitin molecules to substrate proteins, which plays a central role in maintaining cellular homeostasis by regulating protein degradation, trafficking, DNA repair, and signal transduction.

### 2.3 Non-coding RNAs

The discovery of ncRNA dates back to the 1950s and has been an evolving field of research since then (Chen and Kim, 2024). ncRNAs, especially long non-coding RNAs (lncRNAs) and microRNAs (miRNAs), are crucial and important regulators of gene expression and epigenetic changes in both normal physiology and cancer biology. LncRNAs can interact with DNA, particularly purine-rich double-stranded regions, through Hoogsteen base pairing to form RNA–DNA triplex structures, functioning as either transcriptional repressors or activators depending on the required context or function. This structure enables lncRNAs to recruit chromatin modifiers to specific loci, contributing to epigenetic changes and transcriptional regulation (Li, 2023). Moreover, various ncRNAs, including miRNAs and small nucleolar RNAs (snoRNAs), operate as internal molecular signals that regulate key aspects and functions of genes, such as chromatin organization, transcription, RNA splicing, RNA editing, translation, and mRNA degradation, thus shaping cellular differentiation and disease progression (Mattick and Makunin, 2006).

In summary, epigenetic mechanisms comprising DNA methylation, histone modifications, chromatin remodeling, and non-coding RNAs form a complex and interconnected regulatory network that governs gene expression and cellular identity. Disruption of these pathways plays a pivotal role in the initiation and progression of various cancers, including HPV-associated malignancies such as cervical cancer (Da Silva et al., 2021). A deeper understanding of these processes is crucial for future research regarding HPV mediated cancers, including cervical cancer.

## 3 HPV-epigenome interaction

Epigenetics refers to the study of genome regulation through mechanisms beyond the DNA sequence itself, leading to stable changes in gene expression. The landmark discovery was the association of histone complexes with Human Papilloma Virus DNA, as first described by Favre and colleagues in Favre et al. (1977). In recent years, it has been well-established that epigenetics significantly influences various biological processes, including embryonic development, cancer progression, and immune responses. Among the most extensively researched HPV influenced epigenetic changes or modifications are E6/E7-mediated epigenetic disruption, DNA methylation, histone modifications, and non-coding RNAs, which may be influenced by HPV infection (Herceg, 2016; Figure 2).

### 3.1 E6/E7-mediated epigenetic disruption

The high-risk HPV oncoproteins E6 and E7 drive epigenetic reprogramming in host cells to facilitate persistent infection, cellular immortalization, and oncogenic progression. E6 promotes degradation of the tumor suppressor p53, disrupting genomic surveillance and indirectly promoting DNMT upregulation, leading to aberrant DNA methylation patterns. Meanwhile, E7 inactivates pRB, freeing E2F transcription factors to activate targets including EZH2, the catalytic core of the Polycomb Repressive Complex 2 (PRC2), elevating its expression in HPV-infected cells and HPV-positive lesions (Ghiani and Chiocca, 2022). Recent research indicates that E7 binds to and inactivates E2F6 protein, which is associated with polycomb complexes, resulting in epigenetic alterations associated with gene silencing. E7 CD2 domain’s LXCXE motif is seen to be required for pRB inactivation, which is also required for the downregulation of p107 and p130 (Tomaic, 2016).

Despite of the fact that, EZH2, a histone methyltransferase, is overexpressed, in HPV infected cells, H3K27me3 levels, is paradoxically reduced in E6/E7 expressing keratinocytes and cervical precancerous lesions. This unforeseen observation is attributed to the simultaneous induction of the demethylases KDM6A and KDM6B by E7, which remove H3K27me3 and derepress loci such as HOX and p16-INK4A, driving epigenetic reprogramming toward a stem-like, proliferative phenotype. Indeed, depletion of either demethylase in HPV-positive cervical cancer cells inhibits cell proliferation, highlighting their oncogenic relevance (McLaughlin-Drubin et al., 2011).

Additional dysregulation includes phosphorylation of EZH2 (at Ser21) via Akt activation and downregulation of PRC1 component BMI1, further contributes to altered chromatin landscapes and loss of H3K27me3 in E6/E7-expressing cells (Hyland et al., 2011). Transcriptomic data from over 800 human tumors (cervical and head-and-neck) in TCGA confirm elevated expression of EZH2, KDM6A, and KDM6B in HPV-positive cases, along with altered methylation in the CDKN2A locus, reinforcing the mechanistic model in clinical samples (Gameiro et al., 2017).

In sum, the E6/E7 oncoproteins remodel the host epigenome through a coordinated dysregulation of methyl writers and erasers, establishing a unique epigenetic signature characterized by high EZH2 yet low H3K27me3, which helps transcriptional deregulation, loss of differentiation, and malignant transformation. This interaction reveals potential vulnerabilities, as targeting KDM6 demethylases or EZH2 activity may reverse epigenetic reprogramming and inhibit HPV-driven oncogenesis.

### 3.2 DNA methylation alterations

During persistent high-risk HPV (HR-HPV) infection, abnormal DNA methylation events occur in both the host cervical squamous epithelial genome and the HPV viral genome. Increased levels of DNA methylation have been found with histological grades in cervical cancer, and also been found that methylation may increase the cervical intraepithelial neoplasia (CIN) grade I to invasive cervical cancer, while being undetectable in normal cervical epithelium (Laengsri et al., 2018). Epigenetic changes in both the host genome and the viral genome contribute to oncogenesis. In host cells, promoter hypermethylation of tumor suppressor genes such as p16INK4a, RASSF1A, CADM1, DAPK1, and ZNF582 leads to their silencing and promotes uncontrolled cellular proliferation and survival (Laengsri et al., 2018; Boscolo-Rizzo et al., 2017). Concurrently, methylation of the HPV genome, particularly within the long control region (LCR) and E2 binding sites, may alter viral gene expression, including upregulation of E6 and E7 oncoproteins, which in turn inactivate p53 and pRb tumor suppressors (Clarke et al., 2012; Moody and Laimins, 2010; Castro-Oropeza and Pina-Sanchez, 2022). It has been observed that methylation of the long control region (LCR) is more common in uterine cervical cancer (UCC) compared to cervical intraepithelial neoplasia (CIN). Moreover, the hypermethylation of CpG islands within the LCR of HPV16 increases progressively with lesion severity, showing significantly higher levels in invasive cervical cancer (Wang et al., 2017).

Moreover, due to methylation, dysregulations of several genes occur, which affect the immune cell development and their response in the tumor microenvironment (TME) and also affect the prognosis of cervical cancer patients. This may happen due to the methylation of immune-related genes, which affects their expression and dysregulation (Liu et al., 2022). For example, methylation of ERBB3 is oncogenic as RNA and DNA hypermethylation at transcription site genes may change the immune response in cervical cancer (Yang et al., 2022).

HPV16 E7 has been shown to interact with both histone acetyltransferases (HATs) and histone deacetylases (HDACs), thereby influencing chromatin structure and transcriptional activity. In a study by Burgers et al. (2007), E7 was found to interact directly with DNA methyltransferase 1 (DNMT1), enhancing its methyltransferase activity and contributing to aberrant DNA methylation in host cells (Burgers et al., 2007). Additionally, E7 is associated with increased expression of histone H3 lysine 27 (H3K27) demethylases, KDM6A and KDM6B, which remove the repressive H3K27me3 mark, leading to transcriptional activation of oncogenic and stemness-related genes (Basukala and Banks, 2021). Furthermore, recent evidence suggests that E7 enhances the post-translational modification of SETD2, a histone methyltransferase responsible for H3K36me3 deposition, thereby influencing transcriptional regulation and genome integrity (Castro-Oropeza and Pina-Sanchez, 2022). These documented changes in methylation suggest its utility as a biomarker for early detection, triage, and prognosis of cervical cancer.

### 3.3 Histone and chromatin alterations

Several genes were shown to be histone modification markers linked to cervical cancer in a study by Laengsri et al. Although the precise uses of the tumor suppressor gene p53 were not stated, it was examined in cell lines. Histone H3 acetyl K9 (H3K9ac) and Histone H3 trimethyl K4 (H3K4) were analyzed in tissue samples for chromatin remodeling, and their functions were hypothesized to be connected to the prognosis of disease. Retinoic acid receptor beta 2 (RARB2) and p21Cip1/WAF1 were found in cell lines to regulate apoptosis, suggesting possible uses for therapy and treatment monitoring (Laengsri et al., 2018). Without altering the expression of viral oncoproteins E6 and E7, valproate, a histone deacetylase (HDAC) inhibitor, demonstrated an inhibitory effect on cervical cancer cell lines and primary tumors by causing hyperacetylation of p53. Protease inhibitors like bortezomib and HDAC inhibitors like vorinostat or trichostatin A (TSA) also showed a synergistic effect when combined, efficiently eliminating HPV-positive cervical cancer cell lines. These results demonstrate how HDAC inhibitors may be used as epigenetic treatment approaches to treat cervical cancer (Liu et al., 2019).

Through epigenetic regulation, E6 also consecutively expresses hTERT (human telomerase reverse transcriptase, the catalytic unit of the telomerase enzyme). It also regulates methylases and demethylases, raising the H3K4Me3 activation mark and lowering the H3K9Me2 repressive mark (Pal and Kundu, 2019). NFX1-91, a transcriptional repressor, binds to the X box element of the *hTERT* promoter and also interacts with mSin3A, which is a transcriptional co-repressor that recruits HDACs. E6/E6AP complex alters chromatin accessibility at the hTERT by altering histone acetyltransferase (HAT) and histone deacetylase (HDAC) recruitment to the hTERT promoter. E6/E6AP also degrades NFX1-91, which leads to loss of HDAC activity at the promoter, whereas the activity of HAT is increased, which results in an increase in histone acetylation. HPV infection alters the DNA methylation pattern at the hTERT promoter. E6 and E7 of high-risk HPV are also hypermethylated and hypomethylated in certain promoter regions (Katzenellenbogen, 2017).

Moreover, HPV16 E7 also interacts with p300/CBP-associated factor (PCAF) histone acetyltransferase, which in turn downregulates IL-8, which helps the cells infected with HPV evade immune response (Huang and McCance, 2002). Mi2β protein in the presence of HPV E7 influences HDAC1/HDAC2, which regulates cell cycle deregulation and immune evasion through the histone modification process and transcription (Basukala and Banks, 2021). There are several other processes also altered, such as SUMOylation, Acetylation, Phosphorylation, and Ubiquitination. Recent research/literature is discussed below in detail.

### 3.4 SUMOylation

One of the early findings with respect to SUMOylation and HPV comes from the work of D. Rangasamy and K Woytek, where they listed that SUMOylation plays a key role in nuclear transport in turn, regulating E1 replication function, by regulating nuclear replication domains (Rangasamy et al., 2000). Apart from this, recent research has established that SUMOylation has a role in ferroptosis, a controlled type of cell death. The tumor microenvironment frequently contains hypoxia-like circumstances, which greatly increase cervical cancer cells’ resistance to ferroptosis. Hypoxia-like conditions decrease the deSUMOylation enzyme SENP1 while increasing the amounts of KDM4A, SUMO1, and Ubc9. As a result, KDM4A is SUMOylated at the K471 locus, which facilitates its interaction with SUMO1 in the nucleus. These epigenetic modifications increase the expression of GPX4 and SLC7A11, strengthening cervical cancer cells’ resistance to ferroptosis (Xiong et al., 2024).

By influencing protein stability, localization, and activity, SUMOylation plays a pivotal role in HPV-mediated oncogenesis. Sabtani and colleagues showed that SUMOylation, the UBC9/SUMO pathway, facilitates E-cadherin cleavage, which enhances metastasis in HPV associated head and neck cancer (Sabatini et al., 2022). Through the analysis of clinical samples and bioinformatic data, this study demonstrated that SUMOylation of E-cadherin promotes the degradation of its cleaved fragments (Chen et al., 2023). These findings provide new perspectives on the mechanisms underlying E-cadherin downregulation in HPV-positive head and neck cancers, identifying potential avenues for therapeutic intervention. Moreover, the study highlighted the UBC9/SUMO pathway as a key mediator of E-cadherin cleavage in these cancers, underscoring its critical role in HPV-associated tumor progression (Sabatini et al., 2022; Chen et al., 2023).

Chand and team demonstrated that degradation of hADA3, a transcriptional coactivator protein that HPV16 E6 targets, demonstrates a strong correlation between SUMOylation and HPV oncoproteins. The E6AP ubiquitin ligase mediates this connection and promotes the ubiquitin-mediated degradation of hADA3. Interestingly, HPV16 E6 speeds up hADA3’s SUMOylation, destabilizing the protein and preparing it for ubiquitination later on. While overexpression of hADA3 inhibits SiHa cell migration and proliferation, depletion of Ubc9, the only SUMO-conjugating enzyme, stops hADA3 from degrading quickly. These outcomes establish a clear relation in SUMOylation and HPV-mediated carcinogenesis by highlighting the interaction between SUMOylation and ubiquitination in controlling hADA3 stability (Chand et al., 2014).

Moreover, SUMOylation destabilizes the proteins and promotes the nucleocytoplasmic shuttling of the oncogenic transcription factor Forkhead box M1 b (FoxM1 b), a crucial cell cycle regulator. FoxM1b accumulates and overexpresses because of decreased SUMOylation in HPV16 E7-expressing cells. This change highlights the part SUMOylation dysregulation plays in the development of cervical cancer and is ascribed to HPV16 E7’s disruption of the interaction between FoxM1b and Ubc9 (Jaiswal et al., 2015). HPV E6 targets Ubc9 for proteasomal degradation, which has further consequences on the SUMOylation mechanism. Normal cellular processes are hampered when Ubc9 levels are decreased because it alters the host cells’ SUMOylation profile. These modifications imply that changes in SUMOylation brought on by HPV E6 are essential for viral replication and the development of cervical cancer (Heaton et al., 2011). Another level of intricacy is added by the complex interaction between autophagy and SUMOylation in HPV-mediated oncogenesis. An upregulation of Ubc9, an enzyme essential for SUMOylation, accompanies early HPV-driven transformation. Ubc9 breakdown is prevented by HPV E6/E7 proteins, which limit autophagosome-lysosome fusion, even though autophagy normally controls Ubc9 levels. As a result, Ubc9 builds up, strengthening host cells’ resistance to apoptosis, a sign of cancer growth. These results suggest that Ubc9 may be a therapeutic target for cancers linked to HPV (Mattoscio et al., 2017). When taken as a whole, these investigations demonstrate that SUMOylation is an essential regulatory mechanism in cervical cancer. HPV oncoproteins use SUMOylation to promote carcinogenesis by altering important proteins like KDM4A, Sp1, hADA3, and FoxM1b, as well as the SUMOylation mechanism itself. Gaining insight into these processes opens up new approaches that may target SUMOylation in cervical cancer.

### 3.5 Acetylation

In 2018, Elliot J. Androphy and his team highlighted the regulation of HPV replication, mentioning acetylation at the conserved lysine residue of E2 protein (Thomas and Androphy, 2018). Acetylation of lysine residues on host proteins is also essential for advancing the viral life cycle in the context of Human Papillomavirus (HPV) 16. Important regulatory sites are the di-lysines at positions 111 and 112, which are substantially conserved across papillomaviruses. The delicate balance between acetylation and viral replication is demonstrated by the fact that acetylation at K111 promotes viral replication, whereas deacetylation at the same location limits the helicase activity of E1, through P300 protein and by affecting topo 1 recruitment, hence stops replication (Thomas and Androphy, 2018; Thomas and Androphy, 2019). Chromatin alteration is also influenced by HPV genes, including the E6 and E7 oncogenes. Acetylation of histone H3 at lysine 9 (H3K9), a change linked to the stimulation of cell proliferation, survival, and carcinogenesis, results from the activation of E6 and E7 (Feng et al., 2016). Cervical cancer may develop as a result of these oncogenes’ effects on cell proliferation, division, and death. Lysine acetyltransferase (KAT) enzymes catalyze lysine acetylation. By altering histones and non-histones, these enzymes affect gene expression and, consequently, cellular activities via PTMs (Liu et al., 2019).

Histone deacetylases (HDACs), including HDAC2, HDAC3, and HDAC8, also significantly impact various cellular processes. HDAC2 and HDAC3 influence spindle assembly checkpoint signaling, crucial for mitotic regulation. Additionally, HDAC3’s interaction with PIWIL2 affects its role in epigenetic regulation and cell proliferation in cancer. HDAC3’s ability to inhibit the tumor suppressor proteins p53 and p27 further underscores its importance in cellular transformation and tumorigenesis. HDAC8, expressed both in the cytoplasm and nucleolus of HeLa cells, functions as a tubulin deacetylase, with its inhibition stabilizing microtubules and preventing cell migration and mitotic progression. In contrast, the inhibition of HDAC6 leads to the accumulation of connexin 32 (Cx32) in HeLa cells, where the acetylation of lysines in the C-terminal region of Cx32 plays a critical role in regulating cellular proliferation (Alaei et al., 2018; Zhang et al., 2018).

The role of lysine acetyltransferase GCN5 in HPV-induced cell proliferation has also been highlighted. In cells expressing HPV E7 protein, GCN5 is upregulated and promotes acetylation of histones at the E2F1 promoter. This increase in acetylation stabilizes the transcription factor E2F1, facilitating the G1/S transition and promoting cell cycle progression. Knockout of GCN5 disrupts this process, leading to G1-phase arrest. These findings reveal a novel mechanism by which GCN5 contributes to the pathogenesis of cervical cancer by promoting HPV E7-induced proliferation.

In addition, the bromodomain protein BRD2 plays a critical role in acetylation-mediated chromatin remodeling. By binding to acetylated lysines, BRD2 protects acetylated chromatin from HDACs and promotes acetylation spread, thereby enhancing transcriptional activity. BRD4, a member of the same protein family, has been shown to regulate HPV infection, further emphasizing the importance of acetylation in viral gene expression and cellular transformation.

Collectively, these findings underscore the pivotal role of acetylation in cervical cancer biology, particularly in the regulation of histone acetyltransferases, chromatin remodeling, cell proliferation, and viral oncogene expression. The ongoing exploration of acetylation pathways provides valuable insights into potential therapeutic strategies for targeting cervical cancer and other malignancies.

### 3.6 Phosphorylation

Aberrations in phosphorylation signaling pathways are commonly observed in numerous cancers, including cervical cancer, and are often associated with uncontrolled cell growth and survival (Singh et al., 2017), where Human Papillomavirus (HPV) infection is a primary etiological factor. A crucial aspect express oncoproteins E6 and E7 of this process involves phosphorylation-mediated modulation of both viral and host proteins, which alters their activity, stability, and interactions, thereby driving carcinogenesis. HPV E7, for instance, is phosphorylated by host kinases such as casein kinase II (CK2), and this modification regulates its ability to bind and inactivate the retinoblastoma tumor suppressor protein (pRb). Inactivation of pRb releases E2F transcription factors, promoting uncontrolled cell cycle progression. Moreover, phosphorylation affects the stability and oncogenic potential of E7, highlighting phosphorylation as a regulatory switch in HPV-mediated tumorigenesis (Bhattacharjee et al., 2022). Similarly, the E6 oncoprotein targets the p53 tumor suppressor for proteasomal degradation. The phosphorylation status of p53 is crucial for its stability and activation; HPV infection leads to altered phosphorylation patterns that destabilize p53, impairing its role in DNA damage response and apoptosis (Zhang et al., 2015). Beyond direct modification of viral proteins and tumor suppressors, HPV also manipulates host signaling pathways governed by phosphorylation cascades. The phosphatidylinositol 3-kinase (PI3K)/Akt pathway, which controls cell survival and proliferation, is frequently activated in HPV-positive cervical cancer cells through phosphorylation events induced by E7. This activation confers resistance to apoptosis and enhances cellular proliferation, contributing to tumor progression (Wang et al., 2018). Also, USF1/USF2 suppress the activity of hTERT, but when E6 is present, it enhances hTERT transcription by promoting serine 2 phosphorylation of RNA polymerase (McMurray and McCance, 2003). Additionally, mitogen-activated protein kinase (MAPK)/ERK pathway, often modulated via phosphorylation, is also influenced by HPV oncoproteins to promote oncogenic signaling. NF-κB, a key transcription factor involved in inflammation and immune response, is activated by phosphorylation-mediated signaling in HPV-infected cells, facilitating viral persistence and immune evasion (Moody and Laimins, 2010). These phosphorylation-driven alterations in signaling not only enable HPV to bypass cellular checkpoints but also create a microenvironment conducive to malignant transformation. Furthermore, phosphorylation influences HPV’s ability to evade the host immune system by modulating molecules involved in antigen presentation and inflammatory signaling. The crosstalk between HPV oncoproteins and host kinases creates a complex regulatory network that disrupts normal phosphorylation homeostasis, driving cervical carcinogenesis. Understanding these phosphorylation events offers potential therapeutic targets; for example, kinase inhibitors targeting CK2 or PI3K/Akt pathways may counteract HPV-mediated oncogenic signaling. In summary, phosphorylation acts as a critical molecular mechanism by which HPV oncoproteins regulate both viral functions and host cellular pathways, facilitating cervical cancer development. These insights underscore the importance of phosphorylation in HPV-induced carcinogenesis and provide avenues for developing targeted treatments against cervical cancer.

### 3.7 Ubiquitination

The ubiquitin-proteasome system (UPS) is critical in controlling protein turnover and is frequently hijacked by viruses, including Human Papillomavirus (HPV), to facilitate infection, persistence, and oncogenic transformation. In the context of cervical cancer, which is predominantly caused by high-risk HPV types such as HPV16 and HPV18, the viral oncoproteins E6 and E7 manipulate the ubiquitination machinery to degrade tumor suppressor proteins and deregulate cell cycle control. One of the most well-characterized mechanisms involves the HPV E6 protein, which forms a trimeric complex with the cellular E3 ubiquitin ligase E6-AP (UBE3A) and the tumor suppressor protein p53, targeting p53 for ubiquitin-mediated degradation via the proteasome. Loss of functional p53 disrupts cell cycle arrest and apoptosis, allowing cells with DNA damage to proliferate uncontrollably (Scheffner et al., 1993). Similarly, the E7 oncoprotein promotes the ubiquitin-mediated degradation of retinoblastoma protein (pRb), either directly or through induction of specific E3 ligases like cullin 2-containing complexes. This leads to the release of E2F transcription factors, promoting entry into the S-phase of the cell cycle and unregulated cellular proliferation (Huh et al., 2007). Beyond degrading key tumor suppressors, HPV also exploits the UPS to modulate immune responses, enhancing its persistence in host cells. For example, E6 and E7 downregulate components of the antigen presentation machinery by promoting the degradation of MHC class I molecules, reducing immune recognition (Huh et al., 2007).

In addition to targeting host proteins, HPV oncoproteins are themselves regulated by ubiquitination. The stability of E6 and E7 can be modulated by cellular ubiquitin ligases such as HERC1 and UBR4, which fine-tune the levels of viral proteins and influence the progression from precancerous lesions to invasive cancer. Aberrations in the UPS have been observed in HPV-positive cervical cancers, with overexpression of specific deubiquitinating enzymes (DUBs) and E3 ligases contributing to malignant phenotypes and therapy resistance. Therapeutically, the UPS represents a promising target; proteasome inhibitors like bortezomib have shown potential in sensitizing HPV-positive cancer cells to apoptosis by restoring p53 levels and reducing E6/E7 stability (Rampias et al., 2010).

## 4 Non-coding RNA-mediated regulation and alterations

In the HPV-associated carcinogenesis, ncRNAs can play dual roles as oncogenic or tumor-suppressive behavior. Strategies to modulate ncRNA activity, either by inhibiting oncogenic ncRNAs or overexpressing tumor-suppressive ncRNAs, have been proposed as potential therapeutic interventions (Li, 2023). For instance, miR-106a and miR-27a increase radiosensitivity in HPV-positive head and neck squamous cell carcinoma (HNSCC) by targeting RUNX3 and SMG1, respectively, whereas overexpression of miR-125b in HPV-negative cells reduces radiosensitivity by downregulating ICAM2. Additionally, circular RNAs (circRNAs) act as miRNA sponges, modulating the availability of tumor-related miRNAs. For example, circPVT1 suppresses miR-497-5p, promoting cell proliferation in HPV-negative oral squamous cell carcinoma (OSCC) (Bonelli et al., 2021).

HPV oncoproteins E6 and E7 have been shown to upregulate miR-18a, which enhances proliferation and invasion via suppression of STK4, a key tumor suppressor in the Hippo pathway (Morgan et al., 2020). Several tumor suppressor miRNAs (tsmiRs) such as miR-125 and miR-375 inhibit cervical cancer progression by targeting VEGF and MELK, respectively, impairing proliferation, migration, and invasion while promoting apoptosis (Chauhan et al., 2024; Gui et al., 2021). Interestingly, miRNAs like miR-9-5p exhibit dual roles: acting as an oncomiR in HPV16 + SCC cell lines (CaSki, SiHa) by repressing TWIST1 and CDH1, thus promoting epithelial–mesenchymal transition (EMT), but displaying a tumor-suppressive profile in HPV18 + adenocarcinoma cells (HeLa) due to differential regulation of CDH2 (Banuelos-Villegas et al., 2021).

Other oncogenic miRNAs, such as miR-499, enhance tumor aggressiveness by downregulating SOX6 (Chen et al., 2020), while miR-21, a microRNA, fosters the growth, proliferation, invasion, and spread of tumor cells. It accomplishes this by suppressing the tumor suppressor PTEN, which in turn triggers the abnormal activation of the PI3K/Akt signaling pathway. This activation is characterized by an increase in phosphorylated Akt (p-Akt) (Wang et al., 2017; Silvia Lima et al., 2024). Moreover, miR-29a regulates HSP47, a collagen-processing protein, with its downregulation linked to increased cancer cell migration and invasion, implying a role in cervical cancer metastasis. Collectively, these findings underscore the multifaceted roles of ncRNAs in HPV-driven cervical cancer and highlight their potential as diagnostic biomarkers and therapeutic targets, especially for enhancing the efficacy of chemo-radiotherapy (Mattick and Makunin, 2006).

## 5 Clinical aspects of HPV mediated epigenetics in cervical cancer

HPV infection induces several epigenetic modifications in the host tumor suppressor genes and oncogenes, which may serve as biomarkers for cervical cancer screening, triage, and the development of therapeutic targets. Some of the important clinical implications are mentioned below.

### 5.1 Screening potential

Recently, DNA methylation status has been suggested for a triage strategy for HPV cases and for atypical squamous cells of undetermined significance (ASCUS) cases (Burdier et al., 2024). It is well established that HPV infection can develop cervical cancer, and there is a long window for early detection and treatment. So, if any case is found HPV positive, it should go through a second test triage to differentiate the precursor lesions, which may be through persistent infection, and those that do not have infection. This triage strategy to check the DNA methylation of cellular and viral genes has potential for diagnostics for HPV positive patients (Castro-Oropeza and Pina-Sanchez, 2022).

Previous investigations suggested that methylation of the CADM1 and MAL genes may serve as effective biomarkers for identifying cervical intraepithelial neoplasia grades 2 and 3 (CIN2/3), and demonstrating higher sensitivity than traditional cytology-based triage methods (Verhoef et al., 2015). Additionally, epigenetic changes in the form of methylation of the transcription factor ZNF582 have been linked to CIN3 in patients with ASCUS (Burdier et al., 2024), and when it is combined with PAX1, ZNF582 methylation markers have demonstrated potential for detecting high-grade squamous intraepithelial lesions (HSIL) with high sensitivity and specificity (Tian et al., 2017). Apart from this, various genes have been identified with methylation patterns that may serve as promising biomarkers for detecting cervical precursor lesions, such as POUF4 (Castro-Oropeza and Pina-Sanchez, 2022; Pun et al., 2015; Kocsis et al., 2017), ASCL1, LHX8, and ST6GALNAC5 (Verlaat et al., 2018).

Some clinical validations on methylation profiles as diagnostic biomarkers for CIN2, CIN3, and cervical cancer (CC), analyze the methylation status of EPB41L3 and the late regions of HPV types 16, 18, 31, and 33, reporting a sensitivity of 74% and a specificity of 90% (Lorincz et al., 2016; Cook et al., 2019). Recently, a multicenter study reported an impressive 99.8% sensitivity for identifying CIN3 and invasive cervical cancer (Banila et al., 2022). Beyond cervical applications, a panel targeting EPB41L3 along with L1, L2, and E2 regions of HPV16 has been explored for the early detection of oropharyngeal carcinoma, demonstrating a 70% sensitivity and 91% specificity using a non-invasive oral gargle sample (Giuliano et al., 2020).

There are several commercial assays for detecting malignant neoplasms associated with high-risk HPV (HPV-HR), including GynTect^®^ and QIAsure^®^. GynTect^®^ is a molecular test specifically designed to diagnose cervical carcinoma by analyzing the methylation profiles of six biomarkers: ASTN1, ZNF671, DLX1, ITGA4, RXFP3, and SOX17 (Schmitz et al., 2018; Schmitz et al., 2017). In contrast, QIAsure^®^ uses multiplex real-time methylation-specific PCR (qMSP) to detect hypermethylation of FAM19A4 and miR124-2 (Floore et al., 2019). This assay consistently detects cervical cancer across various histological types, including HPV HR-negative cases (Vink et al., 2020). A comparative studies indicate that GynTect^®^ has superior specificity, identifying CIN2 + at 87% versus QIAsure^®^ at 67%, and CIN3 at 84% compared to QIAsure^®^'s 68% (Dippmann et al., 2020). This suggests that GynTect^®^ may be a more reliable choice for early detection of precancerous lesions. In view of these studies, we may use epigenetic changes to enhance the screening methods for early detection of cervical cancer. Some biomarkers are discussed in Table 1.

**Table 1:** DNA methylation biomarkers for cervical cancer screening.

Biomarker	Sample	Clinical relevance	Sensitivity%; specificity%	Reference
CADM1	Cervical scrapes (*N* = 268)	Multiplex methylation analysis of CADM1, MAL, and miR124-2 improves the diagnosis of high-grade lesions and cervical cancer. CADM1 contributes to full detection sensitivity, including certain endometrial malignancies, while MAL and miR124-2 are important markers.	NA; 70	De Strooper et al. (2014)
MAL	Cervical scrapes (*N* = 268)	Multiplex methylation analysis of CADM1, MAL, and miR124-2 improves the diagnosis of high-grade lesions and cervical cancer. CADM1 contributes to full detection sensitivity, including certain endometrial malignancies, while MAL and miR124-2 are important markers.	NA; 70	De Strooper et al. (2014)
miR124-2	Cervical scrapes (*N* = 268)	Multiplex methylation analysis of CADM1, MAL, and miR124-2 improves the diagnosis of high-grade lesions and cervical cancer. CADM1 contributes to full detection sensitivity, including certain endometrial malignancies, while MAL and miR124-2 are important markers.	NA; 70	De Strooper et al. (2014)
ASCL1	HPV-positive cervical scrapes (*N* = 527)	Clinically relevant biomarker with good performance	84.6; 80	Dick et al. (2020) and Verhoef et al. (2022)
LHX8	HPV-positive cervical scrapes (*N* = 527)	Clinically relevant biomarker with good performance	74.9; 80	Dick et al. (2020) and Verhoef et al. (2022)
ST6GALNAC5	HPV-positive cervical scrapes (*N* = 527)	Clinically relevant biomarker with good performance	67.4; 80	Dick et al. (2020) and Verhoef et al. (2022)
GHSR	HPV-positive cervical scrapes (*N* = 527)	Clinically relevant biomarker with good performance	68.6; 80	Dick et al. (2020) and Verhoef et al. (2022)
ZIC1	HPV-positive cervical scrapes (*N* = 527)	Clinically relevant biomarker with good performance	73.7; 80	Dick et al. (2020) and Verhoef et al. (2022)
SST	HPV-positive cervical scrapes (*N* = 527)	Clinically relevant biomarker with good performance	65.7; 80	Dick et al. (2020) and Verhoef et al. (2022)
ANKRD18CP, LHX8 and EPB41L3	hrHPV-positive self-samples (*N* = 304)	ANKRD18CP, LHX8, and EPB41L3 together demonstrated strong diagnostic performance.	training set: 82; 74 test set: 84; 71	de Waard et al. (2023)
ANKRD18CP	hrHPV-positive self-samples (*N* = 304)	Reduced performance on its own, but when combined, it adds value.	47–74; 71	de Waard et al. (2023)
LHX8	hrHPV-positive self-samples (*N* = 304)	Reliable methylation marker that works even with self-samples.	78 (AUC); NA 83–89 in GP-collected samples; NA	de Waard et al. (2023)
EPB41L3	hrHPV-positive self-samples (*N* = 304)	Excellent results in earlier self-sample research; beneficial in panels.	79; 88	de Waard et al. (2023)
LHX8 + ASCL1	hrHPV-positive self-samples (*N* = 304)	Strong alternative; used in non-self-sample setting; not combined in current panel due to redundancy.	83; 82	de Waard et al. (2023)
LHX8, ASCL1 and ST6GALNAC5	hrHPV-positive self-samples (*N* = 304)	Used in selected non-attending women; may not generalize to the general population.	88; 81	de Waard et al. (2023) and Verlaat et al. (2018)
EPB41L3 + JAM3	(*N* = 307)	For CIN2+, methylation of EPB41L3 plus JAM3 exhibited good diagnostic accuracy.	68.7; 86.1	Kong et al. (2023)
SOX14	Tissue specimens and Thinprep cytologic test (*N* = 149)	Potentially useful molecular biomarker for cervical cancer screening and early detection.	diagnosis: 94.12; 86.46 screening:74.42; 81.48	Zhao et al. (2021)
FAM19A4	(*N* = 396)	FAM19A4 methylation is a potential triage tool for women with hrHPV, that can efficiently identify high-grade lesions and cut down on needless colposcopy referrals.	CIN2+: 69.2; 69.9 CIN3+: 75.8; 67.0	De Strooper et al. (2014)
S5	*N* = 710	Shows promise as a triage test for hrHPV-positive women.	90; 49% in high-grade cervical lesions	Albulescu et al. (2021)
ZNF582	*N* = 242	ZNF582 methylation and HPV-16/18 type is positive, colposcopy is recommended	82.43; 76.79	Li et al. (2021)
QCIGISH	105 cytological samples (49 benign, 24 CIN1, 16 CIN3, and 16 malignant)	QCIGISH is a promising triage alternative, based on aberrant expression of GNAS, HM13, and SNU13 imprinted genes.	100% for malignant cases, 93.8% (95% CI, 85.4–100%) for CIN3 and malignant cases; 89.8% (95% CI, 81.3–98.3%) for benign cases 83.6% (95% CI, 75.1–92.1%) for benign and CIN1 cases combined.	Xiao et al. (2024)
SEPT9	*N* = 80	For cervical cancer diagnoses SEPT9 methylation is a promising biomarker	AUC = 85.4; 79.7	
PAX1	*N* = 185	PAX1 have high potential for cervical cancer screening, and more population-based research employing standardized DNA methylation testing is necessary.	78; 91	Lai et al. (2010)
SOX1	*N* = 185	SOX1 DNA methylation shows a lot of promise for cervical cancer screening and calls for more population-based research employing standardized DNA methylation testing.	88; 82	Lai et al. (2010)
LMX1A	*N* = 185	LMX1A DNA methylation shows considerable amounts of potential for cervical cancer screening and demands for more population-based research employing standardized DNA methylation testing.	77; 88	Lai et al. (2010)
NKX6	*N* = 185	Additional population-based research employing standardized DNA methylation testing is necessary since the DNA methylation of NKX6 has significant potential for cervical cancer screening.	93; 97	Lai et al. (2010)
GynTect^®^	*N* = 306	Methylation-specific real-time PCR assay, an additional evidence for the usefulness of methylation markers	CIN3 + 67.7% (95% CI 57.3–77.1%); 88.7% (95% CI 83.7–92.6%).	Schmitz et al. (2017)
Multiplex QMSP	*N* = 402	CADM1, MAL, FAM19A4 and hsa-miR124-2 promoter methylation level was assessed using multiplex PCR.	74; 61	Salta et al. (2021)
CDH6, GATA4 and LHX8	*N* = 152	After MethylCap-seq, genes were identified and validated	73 to 84% sensitivity	Boers et al. (2016)
ZSCAN1, ST6GALNAC5, and KCNIP4	*N* = 152	After MethylCap-seq, genes were identified and validated	>92% Sensitivity;	Boers et al. (2016)
ASCL1/LHX8	*N* = 183	ASCL1/LHX8 showed clinical application to detect CIN3 + in hrHPV-positive women.	76.9%; 74.5%	Verhoef et al. (2022)

### 5.2 Therapeutic potential

The reversible nature of epigenetic alterations gives crucial therapeutic windows for cervical cancer as well as HPV-driven malignancies. Unlike genetic mutations, epigenetic changes such as DNA methylation and histone modifications are dynamic and potentially reversible, making them suitable targets for therapeutic intervention. In HPV-positive cervical cancers, the viral oncoproteins E6 and E7 induce epigenetic silencing of tumor suppressor genes and reprogram host transcription, thus creating a dependency on the altered epigenome that can be exploited therapeutically.

Drugs such as decitabine and azacitidine, which inhibit DNA methyltransferases (DNMTs), have been extensively studied for their ability to demethylate and reactivate silenced tumor suppressor genes. These agents incorporate into DNA and trap DNMTs, leading to hypomethylation and re-expression of epigenetically silenced genes, including those involved in cell cycle regulation and apoptosis (e.g., *p16INK4a*, *DAPK1*) (Hu et al., 2021; Chiappinelli et al., 2015). HDAC inhibitors such as vorinostat (SAHA), belinostat, and romidepsin function by increasing histone acetylation, leading to a more relaxed chromatin structure and transcriptional activation of silenced genes. In cervical cancer, HDAC inhibitors can induce apoptosis and cell cycle arrest, downregulate HPV E6/E7 expression, upregulate tumor suppressor genes (e.g., *p53*, *p21*), and enhance radiosensitivity and chemosensitivity of cervical cancer cells (Lourenco de Freitas et al., 2020; Cheng et al., 2019).

Apart from this, recent studies suggest that epigenetic regulation plays a crucial role in shaping host immune responses during viral infections, including those caused by human papillomavirus (HPV). Studies have shown that viral infections can induce epigenetic alterations in key immune response genes such as AIM2, BST2, BTN3A3, and IL12RB1 (Gasperov et al., 2014). HPV oncoproteins E6, E7, and E5 are known to manipulate the immune system by targeting multiple immune-modulatory pathways. E6 interferes with type I interferon signaling by preventing STAT1 and STAT2 phosphorylation through its interaction with TYK2, impairing IFN-*α*-mediated innate immunity (Castro-Munoz et al., 2022). E7, in turn, interacts with IRF-1 and the Nucleosome Remodeling and Deacetylase (NuRD) complex containing HDAC3, suppressing transcriptional activation of IFN-*β* and other interferon-stimulated genes. Furthermore, E5 and E7 downregulate major histocompatibility complex (MHC) class I expression, hampering antigen presentation to cytotoxic T lymphocytes (Westrich et al., 2017).

There is growing interest in combining epigenetic drugs with immunotherapy. Epigenetic modulation can enhance expression of tumor-associated antigens, upregulate MHC class I molecules, and induce type I interferon responses, thereby increasing immune recognition of HPV-transformed cells. Early-phase clinical trials are exploring the combination of DNMT inhibitors and HDAC inhibitors with PD-1/PD-L1 checkpoint blockade in HPV-associated malignancies, including cervical cancer (Zhou et al., 2021; Chen et al., 2022). Some therapeutic targets and inhibitors and ongoing trials have been listed in Tables 2, 3, respectively.

**Table 2:** Some existing epigenetic therapeutic targets and inhibitors.

Group	Compound	Target	References
HDAC inhibitors	Quercetin	HDAC 2, 4, 7, 8	Lourenco de Freitas et al. (2020); Biswas et al. (2018)
	Trichostatin	HDAC I II	Lourenco de Freitas et al. (2020); Takai and Narahara (2007)
	Caffeic acid	HDAC inhibitor in HeLa	Lourenco de Freitas et al. (2020)
	BML210	HDAC 1, 5, 7 inhibitions in HeLa	Borutinskaite et al. (2006)
	Luotonin A derivavtive	HDAC 1, 2 inhibitors	Lourenco de Freitas et al. (2020)
	Belinostat	HDAC 1 inhibitor	Lourenco de Freitas et al. (2020)
	Romidapsin.	HPV E6 and E7 protein	Lourenco de Freitas et al. (2020) and Łuczak and Jagodzinski (2008)
	Suberoylanilide hydroxamic acid (SAHA)	Elevates Parkin acetylation, increases mitophagy	Sun et al. (2022)
Redox inhibitors	Vitamin E, EGCG and curcumin	MAPKs, Akt/TSC2/mTORC1, Wnt/*β*-Cat, NFkB/IkB/NOX2, HIF/VHL/VEGF pathways; ROS and oxidation of unsaturated fatty acids	Zhang et al. (2023), Zhao et al. (2024), and Sharifi-Rad et al. (2020)
Methylation inhibitors	Hydralazine	APC methylation	Song and Zhang (2009)
	Sodium valproate	Cell cycle G1 arrest and histone methylation	Rocha et al. (2024)
	Alpha-linolenic acid (ALA)	5’ CpG island of DAPK1, CDH1, and RARβ	Ulhe et al. (2025)
Lysine demethylase/ KDM inhibitors	GSK-J4	p16INK4A	McLaughlin-Drubin et al. (2013)
	Zeste 12 (Suz12)	miR-424-5p	Liu et al. (2020)
Kinase inhibitors	MK-0457, BI-847325, PHA-739358, and ENMD-2076	PI3K/AKT/mTOR pathway	Oladipo and Parish (2025)
	Resveratrol	hexokinase-2, pyruvate kinase M2, and lactate dehydrogenase A	Burmeister et al. (2024)
	IMD 0354 (N-(3,5-Bis-trifluoromethylphenyl)-5-chloro-2-hydroxybenzamide)	inhibitor of nuclear factor kappa-B kinase subunit beta’ (IKKβ)	Padash Barmchi et al. (2021)

**Table 3:** Clinical trials exploring epigenetic modulations for HPV.

Current studies	Scope of study	Enrolments	Status	Reference
Study on novel strategies for cervical cancer screening using photoelectric detection and epigenetic procotol (CC-AZ)	Methylation test and colposcopic biopsy, and clinical follow-up based on methylation results	4,200	Not applicable	NCT06866392
TRANSKRIP^®^ plus chemotherapy in recurrent-persistent cervical cancer	To assess survival of patient with TRANSKRIP.	0	Phase 3, interventional	NCT02446652
Hydralazine valproate for cervical cancer	To assess cisplatin topotecan against placebo plus cisplatin topotecan upon progression-free survival.	143	Phase 3, unknown status	NCT00532818
Hydralazine and valproate plus cisplatin chemoradiation in cervical cancer	Inhibitors of DNA methylation and HDAC inhibition synergize the radiation and chemotherapy effects	18	Phase 2, completed	NCT00404326
Omega-3 supplementation in cervix cancer patients undergoing chemoradiotherapy	To assess omega-3 supplementation on body composition, functional capacity, inflammatory profile and quality of life in cervix cancer patients undergoing chemoradiotherapy.	40	Completed, interventional	NCT02779868
A Phase I/ II study of combination immunotherapy for advanced cancers including HPV-Associated malignancies, small bowel, and colon cancers	Histone deacetylase inhibitor (HDAC inhibitor) concomitantly with anti PD-1(L1) therapy help overcome resistance or refractoriness to anti PD-1(L1) therapy alone.	55	Phase 1 Phase 2	NCT04708470
Study of pomalidomide in anal cancer precursors (SPACE)	Immunomodulatory agent pomalidomide in persistent human papillomavirus (HPV) -associated high grade squamous intra-epithelial lesions	26	Phase 2	NCT03113942
Expression and epigenetic silencing of microRNA for predicting therapeutic response and prognosis of HPV-negative HNSCC	Association between miR expression and miR promoter methylation, and the response to therapy and survival	25	Observational, completed	NCT03953443

### 5.3 Treatment resistance

Substantial evidences are available for the therapeutic potential of epigenetic modulation in mitigating treatment resistance including those associated with high-risk human papillomavirus (HPV). Several recent studies have emphasized how targeting epigenetic mechanisms such as DNA methylation and histone modifications can reverse chemoresistance and radio resistance (Wang et al., 2023; Folliero et al., 2023). In cervical cancer, *in vitro* demethylation has been shown to restore the expression of *p73*, a crucial pro-apoptotic gene often silenced in radioresistant cells, thereby sensitizing them to radiation therapy (Liu et al., 2004). Also, Inhibition of histone deacetylases (HDACs) has been reported to enhance the chemosensitivity of HPV-positive tumor cells to agents like cisplatin and taxol by reactivating silenced apoptotic pathways (Ozaki et al., 2008).

Specifically, the combination of DNA methyltransferase inhibitors (DNMTis) with standard chemotherapy has shown synergistic effects, as DNMTis modulate the epigenetic landscape, allowing previously silenced tumor suppressor genes to re-express and respond to cytotoxic stress (Gravina et al., 2010). Moreover, carbon-ion irradiation has been shown to overcome radio resistance induced by disruption of the HPV E2 gene in cervical keratinocytes, suggesting that epigenetic changes in the tumor microenvironment significantly influence therapeutic outcomes (Arians et al., 2019).

These recent findings emphasize on the concept that epigenetic mechanisms not only drive resistance in HPV-associated cancers but also represent actionable therapeutic targets. The reversibility of these changes highlights the translational potential of epigenetic drugs in improving treatment responses and combating resistance, particularly in tumors with HPV etiology.

## 6 Conclusion and future prospects

Understanding cervical cancer and its epigenetic alterations driven by HPV infection is crucial for its management. DNA methylation, histone modifications, and non-coding RNA regulation are reversible and dynamic changes; these changes help the virus persist and contribute to oncogenic transformation. They also offer promising opportunities for diagnosis and treatment. However, more research is needed. In particular, identifying and validating epigenetic signatures at different stages of HPV-associated cervical cancer is essential. Previously, therapeutic agents such as DNA methyltransferase inhibitors (e.g., 5-azacytidine) and histone deacetylase inhibitors (e.g., vorinostat) showed promising results in restoring normal gene function and expression. In this review, we have highlighted how HPV-mediated epigenetic reprogramming affects the function of tumor suppressors and oncogenes and modulates immune evasion mechanisms. These epigenetic changes contribute to disease progression. However, their reversible nature provides a unique therapeutic window. They also offer opportunities to develop biomarkers that can complement current HPV screening and treatment strategies. Such biomarkers may enable early detection, better prognostication, and personalized treatment. Integrating epigenetic biomarkers into cervical cancer care could improve outcomes and support global health efforts. Exploration of HPV-epigenome interactions holds the potential to transform cervical cancer prevention, biomarker discoveries and therapeutic scope.
